# Postpneumonectomy Compression of the Mitral Annulus: Rare Vascular Complication in Sportive Patient

**DOI:** 10.1155/2016/9575894

**Published:** 2016-12-26

**Authors:** David Debeaumont, Susana Bota, Jean-Marc Baste, Marie Bellefleur, Dimitri Stepowski, Florence Vincent, Tristan Bonnevie, Francis-Edouard Gravier, Marie Netchitailo, Catherine Tardif, Alain Boutry, Jean-François Muir, Jérémy Coquart

**Affiliations:** ^1^Unité de Physiologie Respiratoire et Sportive, CHU de Rouen, 76031 Rouen, France; ^2^Service de Pneumologie, CHU de Rouen, 76031 Rouen, France; ^3^Service de Chirurgie Vasculaire et Thoracique, CHU de Rouen, 76031 Rouen, France; ^4^Service de Cardiologie, CHU de Rouen, 76031 Rouen, France; ^5^Service de Radiologie, CHU de Rouen, 76031 Rouen, France; ^6^ADIR Association, Hôpital de Bois-Guillaume, 76230 Bois-Guillaume, France; ^7^UPRES, EA 3830, GRHV, Faculté de Médecine, 76183 Rouen Cedex, France; ^8^CETAPS, EA 3832, UFR STAPS, Université de Rouen, 76821 Mont Saint Aignan, France

## Abstract

Numerous postpneumonectomy complications exist. We present a rare clinical case of postpneumonectomy exertional dyspnea revealing compression of the mitral annulus by the descending aorta. The patient was 42-year-old former smoker with pulmonary emphysema. He has been operated on, in 2012 (i.e., right pneumonectomy). Before the surgery, the patient was a recreational runner. However, after some months, it was difficult for the patient to resume running. Cardiopulmonary exercise testing indicated moderate exercise intolerance with important oxygen desaturation. More interestingly, a decrease of low oxygen pulse was noticed from the first ventilatory threshold with no electrical modification on the electrocardiogram. This decrease was indicative of a decline in stroke volume. The thoracic scan revealed a right pneumonectomy pocket with a liquid abnormal content. Moreover, the mediastinum had shifted toward the pneumonectomy space and the left lung was distended and emphysematous. Echocardiography revealed a major change in the mediastinal anatomy. The mitral annulus was observed to be compressed by the rear wall of the descending aorta. The diagnosis of postpneumonectomy syndrome or platypnea-orthodeoxia syndrome was ruled out in this patient. Mitral annular compression by the descending aorta is rare complication, which must be researched in patients with postpneumonectomy exertional dyspnea.

## 1. Introduction

Postpneumonectomy syndrome is a rare complication caused by a deviation of the mediastinum following pneumonectomy [1]. The mediastinum shifts and rotates into the pneumonectomy space, obstructing the trachea, main bronchus, and esophagus by extrinsic compression and stretching. Vascular complications are rare in this syndrome, generally involving the reopening of a patent foramen ovale or pulmonary artery compression [2]. To our knowledge, only a single case of pulmonary vein compression has been described [3], revealed by exploration of postpneumonectomy exertional dyspnea. We present a rare clinical case of postpneumonectomy exertional dyspnea revealing compression of the mitral annulus by the descending aorta.

## 2. Observation

The patient was 42-year-old former smoker (23 packs/year) with pulmonary emphysema (bilateral emphysematous lesions predominantly in the right upper lobe). The patient (body mass = 67 kg; height = 176 cm; body mass index = 21.6 kg·m^−2^) had been a recreational runner (about 2 hr·wk^−1^; average self-reported speed: 12 km·hr^−1^), but in June 2012 he consulted for chronic cough and general health deterioration. A suspicious mass was detected in the right upper lobe associated with voluminous hilar lymphadenopathy. In July 2012, pulmonary function testing (PFT) indicated normal lung volumes and airflow and moderately reduced alveolar-capillary membrane diffusing capacity (carbon monoxide diffusing capacity: DLCO = 66.4% of predicted) (Table 1). Lymph node exploration by fine needle aspiration and mediastinoscopy was inconclusive and the decision for surgery was made by the multidisciplinary team. The intervention was performed without complication in August 2012, with wedge resection followed by right pneumonectomy under video-assisted thoracoscopy and lymph node dissection based on intraoperative findings. Histopathology confirmed a large cell carcinoma associated with areas of moderately differentiated adenocarcinoma. The pathological stage was pT1bN1M0, IIa. The intervention was followed by four cycles of adjuvant chemotherapy with cisplatin and pemetrexed between September 2012 and February 2013. Follow-up with regular clinical monitoring and computed tomography (CT) scans indicated satisfactory clinical status, despite some right chest pain. The scans showed no disease recurrence, and the right pneumonectomy pocket was visible, with the left lung expanding into the right thorax.

After chemotherapy, it was difficult for the patient to resume running. An exercise rehabilitation program was thus proposed in May 2013, preceded by another PFT and cardiopulmonary exercise testing (CPX). The PFT indicated moderate restriction, with DLCO 48.2% of predicted (Table 1). The CPX was performed on a cycle ergometer (3-min warm-up at 30 W followed by increments of 15 W·min^−1^) and indicated moderate exercise intolerance (the highest possible level of power developed by patient during CPX, called: maximum aerobic power, MAP = 150 W, or 76% of theoretical MAP) with oxygen desaturation (oxygen saturation: SpO_2_, from 98% to 88% at the end of exercise).

Following this assessment, a 5-month home exercise rehabilitation began (3 weekly walking sessions at a heart rate of 120–125 bpm, i.e., about 60% of real MAP). The patient reported following these instructions.

At the end of the program (October 2013), the patient resumed running about 6 hr·wk^−1^ but complained of persistent exercise intolerance and a decline in performance (i.e., average speed: 12 km·h^−1^ versus 7 km·h^−1^, resp., before and after surgery). Therefore, a second CPX was performed in October 2013 on a treadmill (3-min warm-up at 3 km·h^−1^ at 0% incline followed by increments of 1 km·hr^−1^ per minute at a 2% incline) to reproduce as much as possible his running habits. The low ventilatory reserve (VR = 11.4%) and high respiratory exchange ratio (RER = 1.23) and heart rate (HR = 182 bpm) measured at the end of testing confirmed that the test was maximal. The patient's peak oxygen uptake (${\dot{V}O}_{2peak}$) was 31.7 mL·min^−1^·kg^−1^, or 87% of theoretical ${\dot{V}O}_{2peak}$. However, the oxygen (O_2_) pulse kinetics was abnormal, showing a decrease from the first ventilatory threshold (VT_1_) with no electrical modification on the electrocardiogram (from 14 to 13 mL·beat^−1^ between VT_1_ and ${\dot{V}O}_{2peak}$). The persistence of significant desaturation (SpO_2_ from 97 to 85%) related to the pulmonary emphysema was also observed.

Any change in expiratory flow-volume curves was noted. Moreover, any inspiratory stridor or expiratory whistling was found.

**Figure 1:**
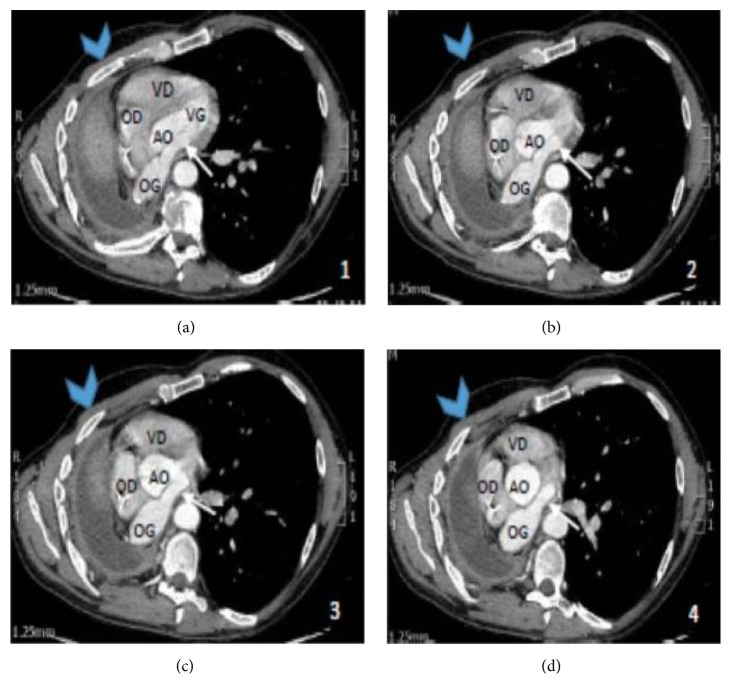
Thoracic computed tomography. VD, right ventricle; VG, left ventricle; OD, right atrium; OG, left atrium; AO, ascending aorta.

The thoracic CT scan (Figure 1) revealed a right pneumonectomy pocket with a liquid abnormal content. Moreover, the mediastinum had shifted toward the pneumonectomy space and the left lung was extremely distended and partially emphysematous, particularly the upper lobe.

An echocardiography at rest and during exercise was performed in December 2013 to determine whether the low O_2_ pulse was indicative of stagnation or decline in stroke volume (SV) at the end of exercise. From these tests, the cardiologist noted a major change in the mediastinal anatomy.

At rest, the mitral annulus was observed to be compressed by the rear wall of the descending aorta. There was no mitral regurgitation and the mitral gradient was not measurable because of the anatomical presentation. The ejection fraction of the left ventricle was 57%. No pulmonary hypertension or pericardial effusion was found.

**Figure 2:**
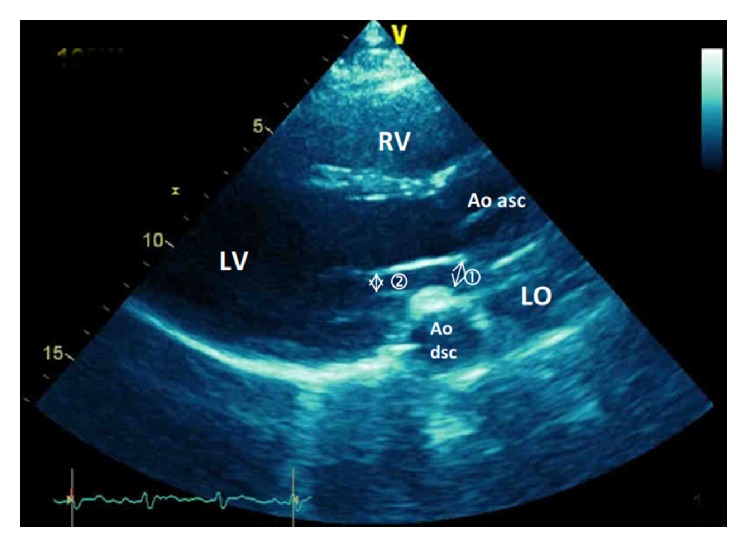
Echocardiography at maximal exercise. RV, right ventricle; LV, left ventricle; LO, left atrium; Ao dsc, descending aorta; Ao asc, ascending aorta; ①, compression of the mitral annulus; ②, compression of the mitral orifice.

At maximum pedaling (i.e., 120 W, Figure 2) on an inclined surface (2-min warm-up at 20 W followed by increments of 20 W·min^−1^), the left and right ventricular kinetics were satisfactory. There was no valvular disease. Systolic pulmonary artery pressure was normal (SPAP = 53 mmHg). The left atrial ejection fraction indicated persistent obstruction. The mitral annulus was narrower than normal, since it was estimated at 12 mm, or reduced by about 40%. The diagnosis of postpneumonectomy syndrome or platypnea-orthodeoxia syndrome was ruled out in this patient (no dyspnea in the upright position and absence of a patent foramen ovale). A change in the mediastinal anatomy had obstructed left ventricular filling through mitral annular compression by the descending aorta.

In the 6 months following these exams, the patient continued to run (6 hr·wk^−1^), and in June 2014 he again underwent exercise echocardiography. This exam confirmed the persistence of a compressed mitral annulus by the descending aorta with no decrease in cardiac output. The patient had nevertheless improved his performance.

Since these last exams, the patient has continued running at an adapted moderate intensity. His exercise tolerance is satisfactory.

## 3. Discussion

Mitral annular compression by the descending aorta is a cardiovascular complication following pneumonectomy that has never been described.

The complications of lung resection are many and have been well described by Jayle and Corbi [4]. The complications after pneumonectomy are especially congestive heart failure, unrecognized prior to pulmonary hypertension and pulmonary embolism [5]. Age and cardiovascular comorbidities are the main risk factors of complications [6], and supraventricular arrhythmias are common postoperatively. Screening for other cardiovascular complications should be performed: heart failure, myocardial infarction, cardiac herniation, and platypnea-orthodeoxia syndrome should be considered in cases of late-onset respiratory failure. Our patient had none of these cardiovascular complications.

Other postoperative complications, particularly bronchopulmonary, should also be sought. For example, pneumonia and atelectasis are common. Postpneumonectomy respiratory failure may evoke acute respiratory distress syndrome or pulmonary edema. Similarly, empyema should raise the suspicion of a bronchial fistula. The rare bronchopulmonary complications include too long stump syndrome, lung granuloma, anastomotic strictures [7] and postpneumonectomy syndrome.

The differential diagnoses in our clinical case were platypnea-orthodeoxia syndrome and postpneumonectomy syndrome. Platypnea-orthodeoxia syndrome is a rare and late complication in connection with a right-left shunt. The latter is related to a preexisting patent foramen ovale whose blood flow is modified by the mediastinal shift. This syndrome usually occurs one year after a right pneumonectomy [8], and the clinical symptomatology is that of late respiratory failure. The patient described in our clinical case had normal blood gases in ambient air in the functional assessments of 2013. Postpneumonectomy syndrome is defined as airway obstruction caused by the rotation of the mediastinum into the pneumonectomy cavity, usually occurring after a right pneumonectomy [9]. This syndrome is rare (0.1%) and late-occurring [4]. Our patient presented no signs for this diagnosis (no ventilatory obstruction on PFT and CT scan, indicating no airway compression).

The underlying pathomechanism of this rare clinical case was a kinking effect of the left lower lobe vein over the descending aorta, caused by the shifting of the heart toward the right side. The kinking of a lobar vein caused by the mediastinal shift after right-sided pneumonectomy can rapidly lead to severe cardiorespiratory failure. This complication is rare and only one clinical case has recently been reported to our knowledge [3]. The difference between this clinical case [3] and our patient concerns the very bad postoperative clinical tolerance and the need for urgent chirurgical intervention in the case reported by Schweiger et al. [3].

Various treatments have been described for complications after pneumonectomy, especially after postpneumonectomy syndrome. A possible treatment for the patients with this syndrome would be the mediastinal repositioning using expandable saline prostheses [2, 10, 11]. However, as the patient has continued running at moderate exercise intensity and as his exercise tolerance is satisfactory, it has not been decided to implant (for the time) expandable saline prostheses.

## 4. Conclusion

In the current manuscript, we present a rare clinical case of postpneumonectomy exertional dyspnea revealing compression of the mitral annulus by the descending aorta. Consequently, mitral annular compression by the descending aorta is rare complication, which must be researched in patients with postpneumonectomy exertional dyspnea.

## Figures and Tables

**Table 1:** Lung function test before and after pneumonectomy.

	Before pneumonectomy (July, 2012)	After pneumonectomy (May, 2013)
Forced vital capacity (FEC in L/%)	6.00/124	3.37/72
Forced expiratory volume in one second (FEV_1_ in L/%)	4.29/112	2.67/70
FEV_1_/CVF (%)	71.5	79.0
Residual volume (L/%)	2.60/131	1.73/85
Total lung capacity (L/%)	8.60/125	5.21/75
Diffusing capacity of the lung for carbon monoxide (DLC_O_ in %)	66.4	48.2
DLC_O_/alveolar volume (%)	62.6	75.0
